# Fertility Regulation in Male Rats by Implemented Tetraazamacrocyclic Compounds of Iron(II): Synthetic, Spectroscopic, and Applied Aspects With Toxicological Screening

**DOI:** 10.1155/BCA/2006/17316

**Published:** 2006-05-22

**Authors:** Ashu Chaudhary, Ran Vir Singh

**Affiliations:** Department of Chemistry, University of Rajasthan, Jaipur 302 004, Rajasthan, India

## Abstract

Antifertility and histopathological investigations were carried out on reproductive organs of male albino rats induced by tetraazamacrocyclic complexes of iron(II). The complexes were synthesized by the template condensation of 1,2-diaminoethane, 1,3-diaminopropane with succinic acid and phthalic acid in 2 : 2 molar ratios which are abbreviated as [Fe(TAML^*n*^)OAc] (*n* = 1 or 2 and TAML^*n*^ represents tetraazamacrocyclic ligand). The complexes have been characterized by elemental analysis, conductivity measurements, IR, and electronic spectra.

## INTRODUCTION

Today, rapidly expanding population and limited sources are thought to be the most pressing global problems. This rapid increase in the world population has multiplied the benefits of economical and technological advancements. Fertility control is very essential for maintaining satisfactory standards in the developing countries. There is an increasing international recognition for the need to control human fecundity. Needless to say there is an immediate need for an inexpensive, safe, and effective as well as universally acceptable contraceptive. For evolution of such an ideal method for control of human fertility, it is necessary that the reproductive process both of male and female need to be more intensively investigated.

The population of India is multiplying at an alarming rate and has almost crossed one billion by this time. Fertility regulation has therefore become the major concern for the people of all the walks of life. The dramatic success of oral contraceptives in women and the lack of pill for man have stimulated research in male fertility control. The development of new and improved contraception agent for men has lagged behind the development of female contraceptives.

The male, an integral part of the family unit, has largely been sidelined by family planners. Currently, efforts are being made to develop a male contraceptive agent, which would inhibit fertility without affecting sex accessory function and libido. In this endeavor, a variety of synthetic compounds have been evaluated in males of laboratory species of mammals [1–5]. The results obtained are also encouraging. Therefore, this approach may form the basis for clinical regulation of male fertility in future. Inorganic compounds have also been investigated and applied for antifertility activity only and have not been screened for toxicological effect [6–9]. In this context, the present communication deals with the synthesis, characterization, and contraceptive efficacy of iron(II) tetraazamacrocyclic complexes. The complexes screened were investigated for their antifertility efficacy at biochemical and histopathological levels.

The tetraazamacrocyclic complexes of iron(II) reported in this paper have been synthesized for the first time. The complexes have been isolated in pure form through crystallization. These show good antifertility activity and may be useful for medicinal purpose. Further, it has been reported that manganese salts (chloride, nitrate, and acetate) cause loss of testicular germ cells in rats and rabbits, and decreased libido and impotency were noted in men occupationally exposed to manganese [10]. Similarly, macrocyclic complexes of manganese(II) and iron(II) exhibit a broad spectrum of antimicrobial, antiinflammatory, analgesic, and antifertility activity [11]. The above discussion has inspired us to synthesize tetraazamacrocyclic complexes of iron(II) and then screen them for their antifertility activity.

## EXPERIMENTS

### Synthesis

#### Materials and methods

The chemicals including succinic acid, phthalic acid (Fluka chemie AG, Industriestrasse, CH 9471, Buchs, Switzerland), 1,2-diaminoethane, 1,3-diaminopropane (Merck Limited India, Shiv Sagar Estate A, Dr, Annie Besant Road, Worli, Mumbai 400018), and Fe(CH_3_COO)_2_ (BDH) were used.

#### Synthesis of 2,5,10,13-tetraoxo-1,6,9,14-tetraazacyclohexa decane iron(II): [Fe(TAML^1^)(OAc)_2_]

The reaction was carried out in 1 : 2 : 2 molar ratios. Fe–(CH_3_COO)_2_ (5 mmol) was dissolved in methanol (25 mL) and cooled in an ice bath. To this solution, taken in a 100 mL round bottom flask, was added 1,2-diaminoethane [corresponding to Fe(CH_3_COO)_2_] in methanol (25 mL). The resulting mixture was stirred for 25–30 h. The solid product was isolated by filtration, repeatedly washed with the same solvent, and dried in vacuo. The compound was recrystallized in benzene and again dried in vacuo.

#### Synthesis of 3,4,12,13-dibenzo-2,5,11,14-tetraoxo-1,6,10,15-tetraazacyclooctadecane iron(II): [Fe(TAML^2^)(OAc)_2_]

The same experimental procedure as for [Fe(TAML^1^)(OAc)_2_] was used. The reagents used for this purpose are 1,3-diaminopropane and phthalic acid.

#### Analytical methods and physical measurements

Nitrogen was estimated by Kjeldahl's method. Iron was estimated gravimetrically. Carbon and hydrogen analyses were performed at Regional Sophisticated Instrumentation Centre of Central Research Drugs Institute, Lucknow. Conductivity measurements were made with a Systronic (Model 305) conductivity bridge in dry dimethylformamide. Molecular weights were determined by the Rast Camphor method. The IR spectra of the solid samples were recorded as KBr discs on a Nicolet Magna FTIR-550 spectrophotometer. Electronic spectra were recorded, in the range 200–600 nm, using methanol as the solvent on a UV-160A Shimadzu spectrophotometer.

### Antifertility activity

In the present investigations, healthy adult male albino rats *(Rattus norvegicus)* each having weight between 200–250 g of proven fertility were used. These were preferred over other laboratory mammals because of their medium size, relatively double nature, easy in handling and maintenance, size, relatively tocile nature, covertly observable sex and libido, and in having a relatively short gestation period of 23–30 days. Animals were regularly checked for any disease and if found infected were isolated and treated. Animals were fed on a diet of rat feed pellets obtained from Hindustan Lever Ltd, Mumbai, India, and soaked gram. Water was provided *ad libitum*.

In these investigations, doses of the compounds mixed in vehicle (olive oil) were given orally with the help of hypodermic syringe having pearl point needle, for 60 days and withdrawal (recovery) for 30 days (Table 1).

The LD_50_ is a statistically derived single dose of a substance that can be expected to cause death in 50% of the animals. In a prohibited analysis method of LD_50_, the selected dose levels should bracket the expected LD_50_ value with at least one dose level higher than the expected LD_50_ but not causing 100% mortality and one dose level below the expected LD_50_ but not causing 0% mortality. Toxicity of the complexes was determined by calculating the LD_50_ values. Symptoms of poisoning and mortality were observed and results of toxicity were analyzed for determination of LD_50_ values of the complexes. On the basis of LD_50_, values the present (only single) dose of the compounds were decided for the experiment.

#### Fertility test

After the completion of the treatment, the fertility test was done. On day 61, the animals were autopsied and blood was extracted from heart. The serum was separated and used for serum biochemistry. Reproductive tissues (testis, epididymis, vas deferens, seminal vesicle, and ventral prostate) and vital organs (liver, kidney, heart, and adrenal) were blotted free of blood, weighed, and used for tissue biochemistry and histology.

#### Body and organ weights

Initial and final body weights of animals were taken before and after the treatment. The mean value of each of the tissues was calculated.

#### Spermatozoa motility and count

The spermatozoa motility was determined according to the method of Prasad et al [12] using WBC counting Neubauer chamber of a hemocytometer and was expressed as million spermatozoa/mL suspension.

#### Biochemical studies

*Protein* was estimated by Lowry et al's [13] method. *Sialic acid* was estimated by the procedure given by Warren [14]. Cholesterol was done as per the method of Zlatkis et al [15]. *Glycogen* was estimated by the method of Montgomery [16]. *Fructose* was done by the method of Foreman et al [17]. *Ascorbic acid* was estimated by the method of Roe and Kuether [18]. *Acid phosphatase* and *alkaline phosphatase* were measured by the method of Fiske and Subbarow [19].

## RESULTS AND DISCUSSION

### Synthetic

The complexes are colored solids and soluble in most of the organic solvents like methanol, benzene, dichloromethane, tetrahydrofuran, and dimethylformamide. Their nonelectrolytic nature was confirmed by low molar conductance values 15–23 Ohm^−1^cm^2^ mol^−1^. Physical properties and analytical data of the complexes are given in Table 2.

#### IR spectra

The IR spectra of the starting materials and their iron complexes were recorded and their comparative study confirmed the formation of macrocyclic complexes with the proposed coordination pattern. The bands observed in the region 3420−3531 cm^−1^ attributed to −NH_2_ of amino and 2500−2590 cm^−1^ attributed to −OH of the carboxylic acid. Both of these bands (−NH_2_ and −OH) disappeared in the complexes, confirming the cyclization. A single sharp band is observed at 3132 cm^−1^ for [Fe(TAML^1^)(CH_3_COO)_2_] and at 3251 cm^−1^ for [Fe(TAML^2^)(CH_3_COO)_2_] due to *ν*(N−H) of the amide group. The appearance of four bands at 1649, 1565, 1271, and 638 cm^−1^ for [Fe(TAML^1^)(CH_3_COO)_2_], and 1687, 1561, 1249, and 670 cm^−1^ for [Fe(TAML^2^)(CH_3_COO)_2_] are due to the amide I, amide II, amide III, and amide IV vibrations, respectively. The bands at 430 cm^−1^ for [Fe(TAML^1^)(CH_3_COO)_2_] and 442 cm^−1^ for [Fe(TAML^2^)(CH_3_COO)_2_] in the spectra of the complexes may be assigned to (Fe−N) stretching vibrations. The absorption bands observed in the regions 2880−2910 and 1420−1440 cm^−1^ in the complexes may be reasonably assigned to the C−H stretching and bending vibrational modes.

To distinguish between these structures (enolic and amide), we have synthesized the free ligands and recorded their IR spectra. Surprisingly, there were no bands for the azomethine group (>C=N) in the IR spectra of the complexes. This clearly indicated that the amide structure is the real structure in this case and not the enolic structure. The *ν*_asym_ and *ν*_asy_ OCO of the acetate group appear at 1480 and 1330 cm^−1^ (Δ*ν*150 cm^−1^) suggesting the bonding mode of acetate group [20]. A sharp band observed at 482 and 487 cm^−1^ ascribed to the Fe−O stretching vibrations in the complexes [Fe(TAML^1^)(CH_3_COO)_2_] and [Fe(TAML^2^)(CH_3_COO)_2_], respectively. Similar results have also been confirmed and reported by other authors [21].

#### Electronic spectra

A weak intensity band exhibited at 11904 and 11278 cm^−1^ for [Fe(TAML^1^)(CH_3_COO)_2_] and [Fe(TAML^2^)(CH_3_COO)_2_], respectively, is assigned to the ^5^T_2*g*_ → ^5^E_*g*_, transitions consistent with an octahedral geometry for iron(II) complexes. These assignments are also in tune with the results of Lobana et al [22] and Chaudhary et al [23] for high-spin octahedral iron(II) system.

**Figure 1:**
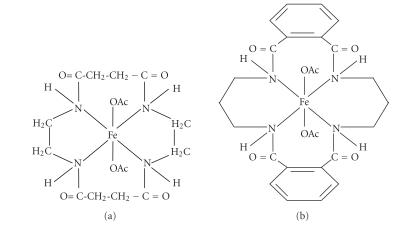
Proposed structures of the complexes.

On the basis of the above spectral studies, the derivatives have been assigned the following structures with hexacoordinated iron atom (Figure 1).

### Antifertility activity

#### Body and organ weights

The data revealed that the body weights of rats were not much altered after the treatment of [Fe(TAML^1^)(CH_3_COO)_2_] and [Fe(TAML^2^)(CH_3_COO)_2_], however, in both the treated groups, a general decrease in the reproductive organ weights was observed in relation to the control. A significant reduction was observed in weights of testis (*P* < 0.01), epididymis (*p* < 0.001), seminal vesicle (*p* < 0.01) and ventral prostate (*p* < 0.001) in [Fe(TAML^1^)(CH_3_COO)_2_]  treated animals. The treatment of [Fe(TAML^2^)(CH_3_COO)_2_] also resulted in a significant decrease in the weights of testis (*p* < 0.001), epididymis (*P* < 0.001), vas deferens (*p* < 0.01), seminal vesicle (*p* < 0.001), and ventral prostate (*p* < 0.01).

After the withdrawal of both of the compounds, the results favour that [Fe(TAML^1^)(CH_3_COO)_2_] was comparatively better in treatment. The vital organs data (Table 3) also show similar results as reported for reproductive organs in Table 4.

#### Spermatozoa motility and count

The spermatozoa motility of [Fe(TAML^1^)(CH_3_COO)_2_] and [Fe(TAML^2^)(CH_3_COO)_2_] treated rats showed that the sperms were sluggishly motile without any forward progression. The motility percent after [Fe(TAML^1^)(CH_3_COO)_2_] treatment declined significantly (*p* < 0.001). Following withdrawal of the treatment, the sperms were actively motile showing forward progression and the sperm motility percentage of the above groups recovered completely to normal level (Table 5). The sperm density of cauda epididymis and testicular sperm density diminished significantly (*p* < 0.001) in [Fe(TAML^1^)(CH_3_COO)_2_] and [Fe(TAML^2^)(CH_3_COO)_2_] treated rats and restored partially following 30 days withdrawal in both the groups (Table 5).

#### Fertility test

Normal proestrous females after mating with the males of proven fertility deliver on average 8−10 pups per female. The treated animals exhibited normal libido and mating behaviour, however, in [Fe(TAML^1^)(CH_3_COO)_2_] treated animals, the fertility test gave 90% negative fertility rate and in [Fe(TAML^2^)(CH_3_COO)_2_] treated group, the fertility test was 72% negative. Following withdrawal of the drug, 25% recovery in fertility rate was observed in [Fe(TAML^1^)(CH_3_COO)_2_] treated group of the animals, whereas 42% recovery in fertility rate was observed in the animals treated with [Fe(TAML^2^)(CH_3_COO)_2_] (Table 5).

#### Biochemical changes

*Proteins*

The protein concentration of the reproductive organs was lowered after the treatment of [Fe(TAML^1^)(CH_3_COO)_2_] as compared to the control which was brought to the normal level in testis and cauda epididymis after the withdrawal of the drug (Table 6). A significant reduction in the protein contents of testis (*p* < 0.001), cauda epididymis (*p* < 0.01), and ventral prostate (*p* < 0.001) was observed following the treatment of [Fe(TAML^1^)(CH_3_COO)_2_]. The above changed parameters were restored completely to normal in all the organs after the withdrawal of the drug (Table 6).

*Sialic acid*

The testicular and cauda epididymal sialic acid concentration of [Fe(TAML^1^)(CH_3_COO)_2_] and [Fe(TAML^2^)(CH_3_COO)_2_] treated animals decreased significantly (*p* < 0.001). Withdrawal period of 30 days did not bring much change in the above content (Table 6).

*Fructose*

The fructose concentration of seminal vesicle was diminished significantly (*p* < 0.001) in [Fe(TAML^1^)(CH_3_COO)_2_] and [Fe(TAML^2^)(CH_3_COO)_2_] treated animals, which was recovered to the normal level after the withdrawal of the drug (Table 6).

*Cholesterol*

The cholesterol and testicular cholesterol content of adrenal increased significantly in [Fe(TAML^1^)(CH_3_COO)_2_] and [Fe(TAML^2^)(CH_3_COO)_2_] treated animals in comparison to the control. Withdrawal of the treatment attained the normal cholesterol and testicular cholesterol level (Table 6).

*Glycogen*

The glycogen concentration of testis was reduced in [Fe(TAML^1^)(CH_3_COO)_2_] treated animals as compared to the control, whereas an increase was observed in the testicular glycogen level in [Fe(TAML^2^)(CH_3_COO)_2_] treated group. The above altered parameter was restored completely after the withdrawal of the treatments (Table 7).

[Fe(TAML^1^)(CH_3_COO)_2_] treated group also resulted in a significant reduction in the liver glycogen concentration (*p* < 0.001), which was brought to normal after the withdrawal of the treatment. On the contrary, an increase in the concentration of liver glycogen was evident following the withdrawal (Table 7).

*Ascorbic acid*

Treatment with both the groups resulted in reduction in the ascorbic acid concentration of testis. This decrease was more significant (*p* < 0.001) in [Fe(TAML^1^)(CH_3_COO)_2_] treated group of rats. A decline in the ascorbic acid concentration was also noticed in cauda epididymis in both the treated groups.

The decrease was more significant (*p* < 0.01) in [Fe(TAML^1^)(CH_3_COO)_2_]. The above altered parameter was brought to normal following 30 days withdrawal of the drug. (Table 7).

*Acid and alkaline phosphatase*

A significant decrease in the acid phosphatase content (*p* < 0.01) of ventral prostate was observed in the [Fe(TAML^1^)(CH_3_COO)_2_] and [Fe(TAML^2^)(CH_3_COO)_2_] treated groups, which was completely restored to normal after the withdrawal in [Fe(TAML^1^)(CH_3_COO)_2_]. However, a partial recovery was observed in [Fe(TAML^1^)(CH_3_COO)_2_] treated group of animals (Table 7). Similar reduction was also evidenced in the concentration of alkaline phosphatase content (*p* < 0.05) of ventral prostate of both the groups.

*Haematology and serum biochemistry*

Haemoglobin and haematocrit values remained unaltered, indicating normal blood physiology of all the groups of animals. However, treatment with [Fe(TAML^1^)(CH_3_COO)_2_] resulted in increased serum cholesterol concentration (*p* < 0.01). The concentrations of VLDL, triglycerides, albumin and A/G ratio were decreased. Treatment with [Fe(TAML^1^)(CH_3_COO)_2_] also caused a significant increase in the serum cholesterol concentration (*p* < 0.01) while concentration of VLDL (*p* < 0.05), triglycerides (*p* < 0.05), albumin, and A/G ratio decreased. The protein and glubulin concentration of serum was increased to some extent in both the treated groups of the animals.

The changes which have been found in the investigating parameters are found to be restoring to the normal partially or completely ultimately recovered. The results (Table 8) indicated that haemoglobin, haematocrit, protein, and albumin were restored completely following withdrawal of the test compounds. However, VLDL, serum, A/G ratio, and glubulin were restored partially after 30 days of the stoppage of the compounds administration.

*Serum testosterone*

The ELISA test (Table 9) revealed a significant reduction (*p* < 0.05) in circulating serum testosterone in both the treated groups. Withdrawal of the drug for 30 days restored the level of serum testosterone completely in [Fe(TAML^1^)(CH_3_COO)_2_] withdrawal group of rats while partial recovery was observed in [Fe(TAML^2^)(CH_3_COO)_2_] withdrawal group of animals.

*Histocytometry*

The histocytometric data of [Fe(TAML^1^)(CH_3_COO)_2_] treated rats revealed a decrease in the seminiferous tubule diameters, germinal epithelial cell height (*p* < 0.001), and Leydig cell diameter (*p* < 0.001) of testis along with a significant decrease in the epithelial cell height (*p* < 0.001) and muscle layer thickness (*p* < 0.001) of cauda epididymis. The epithelial cell height of seminal vesicle also decreased as compared to the control. A significant decrease was noticed in the epithelial cell height (*p* < 0.05) of ventral prostate and in the muscle layer thickness and epithelial cell height (*p* < 0.05) of vas deferens. The above altered parameters were restored following the withdrawal of the treatment (Table 10).

In [Fe(TAML^1^)(CH_3_COO)_2_] treated rats, a significant decrease was noticed in testicular seminiferous tubule diameter (*p* < 0.001), germinal epithelial cell height (*p* < 0.001), and Leydig cell diameter (*p* < 0.01). The epithelial cell height of cauda epididymis decreased significantly (*p* < 0.001) with a decrease in muscle layer thickness. A significant decrease in the epithelial cell height of seminal vesicle (*p* < 0.001) and ventral prostate (*p* < 0.001) was observed. The muscle layer thickness and epithelial cell height of vas deferens were also decreased. Withdrawal of the drug regained the normal levels (Table 11).

*Discussion*

In the present study, the results indicated that the weights of the body and vital organs were not affected after the treatment suggesting that the complexes have no side effect or toxicological effect and maintained normal physiology of the animals throughout the experiment.

- NJ Chinoy and MR Chinoy [24] reported that the structural and functional integrity of the reproductive organs depends on the circulating level of the androgen, and any small change in the androgen level results in the reduction in the weights of the reproductive organs. In the present studies, decline in the circulating levels of testosterone was observed in [Fe(TAML^1^)(CH_3_COO)_2_] and [Fe(TAML^2^)(CH_3_COO)_2_] treated animals leading to a decrease in the organ weights and androgen-dependent parameters.
- The reduction in the weight of the testis after the treatment in the present study related to the loss of spermatozoa and spermatids, which make up a substantial proportion of testicular volume, by the same token, as a consequence of disruption of spermatogenesis [25–27].
- The suppressed epididymal activity as evidenced by the loss of the weight and histological alterations might be due to antigonadotropic activity of the so-termed antiandrogenic/estrogenic substances which lower the ABP production by the sertoli cell and androgen synthesis by the Leydig cells, thereby resulting in reduced levels of androgen available to the epididymis for functional maintenance. The requirement of relatively higher androgen threshold in the epididymis has already been established by Gupta et al [28].
- Reduction in the weights of seminal vesicles, ventral prostate, and vas deferens, also reflects an interference with androgen output. Bhiwgade et al [29] have already correlated the reduction in the weights of sex accessories, particularly of the prostate, following treatment of cyproterone acetate. Antiandrogens are known to depress the uptake of testosterone in the prostate and reduce binding of androgen to the hormonal receptors by a process of competitive inhibition as reported by Sharma and Jacob [30].
- The sperm density and sperm motility have a direct relationship to the fertility [31]. In the present study, sperm motility and density of testis and cauda epididymis after [Fe(TAML^1^)(CH_3_COO)_2_] and [Fe(TAML^2^)(CH_3_COO)_2_] treatments were significantly declined. Rao [32] has reported declined sperm motility, resulting into decreased fertility.
  Immotile or sluggishly motile spermatozoa cannot penetrate the cervical mucus, thus fail to fertilize the ova [33]. During the present course of the study, 90% negative fertility rate was observed after [Fe(TAML^1^)(CH_3_COO)_2_] treatment, while it was 72% negative in the [Fe(TAML^2^)(CH_3_COO)_2_] treated animals. It appears worthwhile to conclude that reduction in the sperm counts and motility recorded in treated animals substantiates the antifertility activity. Further, alteration in the various androgen-dependent biochemical parameters that in turn altered the internal milieu of the epididymis [34] may also be responsible for the negative fertility test. Withdrawal for 30 days in the groups treated with [Fe(TAML^1^)(CH_3_COO)_2_] and [Fe(TAML^2^)(CH_3_COO)_2_] showed significant recovery in the fertility rate, indicating that the effects brought about the synthetic compounds were reversible.
- Protein is involved in the alteration of almost every physiological system and the total protein run parallel to the growth and is sensitive to estrogen and androgen, respectively, reported by Davis et al [35]. Jones [36] has reported that protein level is directly correlated with the secretory activity of the epididymis, which in turn depends on the androgen levels.
  In the present investigations, the reduction in the protein concentration by the treatment may be attributed to the reduction in secretory activity because of the androgen deprivation effect. Kamal et al [4] and Mali et al [37] also reported similar results for the male albino rats.
- Nag et al [38] have reported that an optimal level of sialic acid in reproductive organs is essential for functional integrity of spermatozoa. Our observations are in consonance with those of Gupta and Ahmed [39] who have reported a decrease in sialic acid concentration of testis, cauda epididymis, seminal vesicle, and ventral prostate to albino rats. Bhargava [40] reported decrease in the testicular sialic acid concentration due to the antispermatogenic activity.
- Mammalian cells require cholesterol which plays an important role in acting as precursor molecule in the synthesis of steriod hormone [41]. The requirement of cholesterol for normal activity of testicular glands has been well established by Biswas, Deb, and Johnson [42, 43]. Androgens are synthesized from cholesterol, but increased concentration may result in the reduction of fertility [44].
  After the treatment with the effective doses of both the compounds, there was a significant increase in the testicular and adrenal cholesterol concentration which is in consonance with the results of Kamal et al [4], suggesting that the increased testicular cholesterol concentration may be correlated with its nonutilization by the system leading to a fall in circulatory androgen in rats due to the antiandrogenic activity.
- Baijal et al [45] proposed that glycogen might represent a source of nourishment for the spermatozoa during its development and maturation. The glycogen level declined in testis and liver of the rats treatment with [Fe(TAML^1^)(CH_3_COO)_2_]. Kamal et al [4] have reported decreased concentration of glycogen in the rats due to its androgen dependence, leading to various disorders in testis.
  In contrast, treatment with [Fe(TAML^2^)(CH_3_COO)_2_] caused a nonsignificant increase in the concentration of glycogen of both the liver and testis. The same case has been reported by Ewing et al [46]. Increased carbohydrate utilization has been considered due to androgenic stimulation suggesting a direct correlation between testosterone secretion rate and glucose uptake by the isolated and perfused rabbit testis as reported by Ewing et al [46].
- After treatment with effective doses of [Fe(TAML^1^)(CH_3_COO)_2_] and [Fe(TAML^2^)(CH_3_COO)_2_], there was a decrease in the testicular and cauda epididymal ascorbic acid concentration, which is in consonance with that of Chaterjee et al [47] who reported hypofunctioning of testis and the degeneration of the germinal epithelium due to vitamin C deficiency.
- Decline in acid phosphate level might be due to antiandrogenic effect or might be due to suppressed secretory function of the prostate [28, 32, 44]. The acid phosphate values during present course of the study concur with the findings of Jacob et al [48].
  Administration of the test substances resulted in a significant reduction in the alkaline phosphate content of the ventral prostate. It is evident that this decrease was a likely consequence of spermatogenic arrest as a result of suppression of androgenesis. This observation concurs with the findings of others also [4, 44, 48], who reported a general decline in the alkaline phosphate content of the various reproductive and accessory organs of the male rats after androgen-estrogen therapy.

## Figures and Tables

**Table 1:** Treatment and autopsy days for iron(II) complexes.

Group	Experimental protocol		
	Number of	Treatment	Autopsy
	animals used	days	day

Control	5	—	61st
[Fe(TAML^1^)(CH_3_COO)_2_]	5	60	61st
Withdrawal	5	60 to 30	91st
[Fe(TAML^2^)(CH_3_COO)_2_]	5	60	61st
Withdrawal	5	60 + 30	91st

**Table 2:** Physical properties and analytical data of iron(II) complexes.

Compound	Color and MP (°C)	Yield (%)	Analysis % found (calcd.)				Mol. wt. found (calcd.)
			C	H	N	Fe	

[Fe(TAML^1^)(CH_3_COO)_2_]	Brown	63	41.81	5.62	11.28	11.74	439
	115		(41.96)	(5.72)	(12.23)	(12.19)	(458)

[Fe(TAML^2^)(CH_3_COO)_2_]	Brown	67	53.50	5.10	8.78	9.15	559
	129		(53.66)	(5.20)	(9.63)	(9.60)	(582)

**Table 3:** Vital organ weight (mg/100 g body wt.) in [Fe(TAML^1^)(CH_3_COO)_2_] and [Fe(TAML^2^)(CH_3_COO)_2_] treated and withdrawal group of rats. Values are mean ± SE.

Group	Vital organ (mg)			
	Liver	Heart	Kidney	Adrenal

Control	3038.60 ± 180.20	254.70 ± 8.39	288.08 ± 4.35	9.40 ± 0.40
[Fe(TAML^1^)(CH_3_COO)_2_]	3220.54 ± 110.42	272.09 ± 6.54	330.20 ± 17.25	12.02 ± 0.60
Withdrawal (30 days)	3550.29 ± 140.39	335.70 ± 7.45	340.55 ± 7.54	13.95 ± 0.89
[Fe(TAML^2^)(CH_3_COO)_2_]	2579.50^NS^ ± 360.50	255.70 ± 5.90	255.60 ± 4.47	11.78 ± 0.52
Withdrawal (30 days)	4452.86 ± 130.23	346.60 ± 18.95	348.10 ± 20.02	7.99 ± 0.73

NS = not significant

**Table 4:** Body (g) and reproductive organ weights (mg/100 g body wt.) in [Fe(TAML^1^)(CH_3_COO)_2_] and [Fe(TAML^2^)(CH_3_COO)_2_] treated and withdrawal group of rats. Values are mean ± SE.

Group	Body Wt. (g)		Reproductive organ (mg)				
	Initial	Final	Testis	Epididymis	Vas deferens	Seminal vesicle	Ventral prostate

Control	202.50 ± 5.45	212.50 ± 4.51	641.09 ± 12.50	243.13 ± 4.86	42.05 ± 1.50	207.21 ± 8.33	123.92 ± 3.82
[Fe(TAML^1^)(CH_3_COO)_2_]	163.60 ± 18.00	194.00 ± 16.05	541.16^b^ ± 25.80	201.37^b^ ± 5.70	40.90 ± 0.82	147.67^b^ ± 13.67	95.40^a^ ± 1.32
Withdrawal (30 days)	210.72 ± 18.62	240.27 ± 18.62	505.16 ± 20.02	184.83 ± 7.12	50.40 ± 2.76	138.60 ± 5.48	180.79 ± 6.96
[Fe(TAML^2^)(CH_3_COO)_2_]	285.20 ± 3.30	255.20 ± 1.87	540.35^a^ ± 16.10	160.84^a^ ± 9.19	35.25^b^ ± 0.345	128.07^a^ ± 7.89	96.39^b^ ± 5.25
Withdrawal (30 days)	266.00 ± 9.85	285.40 ± 4.69	404.65 ± 10.08	142.98 ± 0.69	47.98 ± 0.69	106.44 ± 6.99	105.96 ± 2.89

a = *p* < 0.001, b = *p* < 0.01.

**Table 5:** Sperm motility, sperm density and fertility rate of control, [Fe(TAML^1^)(CH_3_COO)_2_] and [Fe(TAML^2^)(CH_3_COO)_2_] treated and withdrawal group of rats. Values are mean ± SE.

Group	Sperm motility (%)	Sperm density (million/mL)		Fertility (%)
		Cauda epididymis	Testis	

Control	60.79 ± 1.29	53.54 ± 0.38	5.29 ± 0.87	100% +ve
[Fe(TAML^1^)(CH_3_COO)_2_]	25.55 ± 1.59^a^	13.54 ± 2.50^a^	1.30 ± 0.12^a^	90% −ve
Withdrawal (30 days)	62.95 ± 4.57	20.55 ± 4.93	2.25 ± 0.35	25% +ve
[Fe(TAML^2^)(CH_3_COO)_2_]	36.90 ± 2.45^a^	19.99 ± 2.98^a^	1.28 ± 0.28^a^	72.0% −ve
Withdrawal (30 days)	67.98 ± 1.24	20.50 ± 3.54	1.35 ± 3.54	42.0% +ve

a = *p* < 0.001, b = *p* < 0.01.

**Table 6:** Protein, sialic acid, fructose and cholesterol concentration of control, and [Fe(TAML^1^)(CH_3_COO)_2_] and [Fe(TAML^2^)(CH_3_COO)_2_] treated and withdrawal group of rats. Values are mean ± SE.

		Group				
		
		Control	[Fe(TAML^1^)(CH_3_COO)_2_]	Withdrawal (30 days)	[Fe(TAML^2^)(CH_3_COO)_2_]	Withdrawal (30 days)

Protein (mg/g)	Testis	85.04 ± 4.30	75.09 ± 5.89	107.70 ± 8.88	32.57^a^ ± 4.57	80.45 ± 1.40
	Cauda epididymis	56.40 ± 2.70	50.24 ± 4.23	65.10 ± 7.23	42.88^b^ ± 1.29	50.99 ± 4.20
	Ventral prostate	130.09 ± 3.10	124.48 ± 9.64	120.99 ± 15.18	83.09^a^ ± 2.50	144.09 ± 1.26

Sialic acid (mg/g)	Testis	1.18 ± 0.05	0.65^a^0.03	0.72 ± 0.03	0.83^a^ ± 0.015	0.79 ± 0.03
	Cauda epididymis	0.95 ± 0.007	0.56^a^ ± 0.007	0.71 ± 0.02	0.82^a^ ± 0.029	0.83 ± 0.006

Cholesterol (mg/g)	Adrenal	14.50 ± 0.30	22.49^a^ ± 1.58	18.25 ± 0.07	27.19^a^ ± 1.75	10.88 ± 0.40
	Testis	6.13 ± 0.30	15.99^a^ ± 1.20	7.82 ± 0.09	7.54 ± 0.90	7.20 ± 0.10

Fructose (mg/g)	Seminal vesicle	0.25 ± 0.014	0.13^a^ ± 0.004	0.22 ± 0.012	0.17^a^ ± 0.003	0.28 ± 0.028

a = *p* < 0.001, b = *p* < 0.01.

**Table 7:** Concentration of glycogen, ascorbic acid, acid phosphate and alkaline phosphate of control [Fe(TAML^1^)(CH_3_COO)_2_], and [Fe(TAML^2^)(CH_3_COO)_2_] treated and withdrawal group of rats. Values are mean ± SE.

					Acid phosphatase	Alkaline
Group	Glycogen (mg/g)		Ascorbic acid (mg/g)		(mg/g/h)	phosphatase
	Testis	Liver	Testis	Cauda epididymis	Ventral prostate	Ventral prostate

Control	3.50 ± 0.27	22.92 ± 0.85	0.17 ± 0.002	0.15 ± 0.004	5.45 ± 0.42	2.21 ± 0.19
[Fe(TAML^1^)(CH_3_COO)_2_]	3.35 ± 0.19	10.17 ± 0.77^a^	0.13 ± 0.004	0.12^b^ ± 0.002	2.19^b^ ± 0.017	1.39^c^ ± 0.16
Withdrawal (30 days)	3.69 ± 0.29	20.88 ± 0.22	0.12^b^ ± 0.002	0.15 ± 0.006	6.23 ± 0.26	2.50 ± 0.29
[Fe(TAML^2^)(CH_3_COO)_2_]	4.38 ± 0.34	23.19 ± 0.32	0.11^b^ ± 0.003	0.12^b^ ± 0.004	2.25 ± 0.23	1.57^c^ ± 0.19
Withdrawal (30 days)	3.18 ± 0.46	11.97 ± 0.77	0.17 ± 0.006	0.19 ± 0.002	3.87 ± 0.49	1.23 ± 0.25

a = *p* < 0.001, b = *p* < 0.01 and c = *p* < 0.05.

**Table 8:** Haematology and serum biochemistry of control, [Fe(TAML^1^)(CH_3_COO)_2_] and [Fe(TAML^2^)(CH_3_COO)_2_] treated and withdrawal groups of rats. Values are mean ± SE, VLDL represents very low density lipoproteins, Tg denotes triglycerides, A/G ratio denotes albumin/globulin ratio.

		Group				
		
		Control	[Fe(TAML^1^)(CH_3_COO)_2_]	Withdrawal (30 days)	[Fe(TAML^2^)(CH_3_COO)_2_]	Withdrawal (30 days)

Blood	Haemoglobin (%)	13.97 ± 0.04	13.23 ± 0.16	13.74 ± 0.04	13.54 ± 0.14	13.37 ± 0.30
	Haematocrit (%)	47.08 ± 0.39	44.89 ± 0.40	45.76 ± 0.39	46.29 ± 0.20	46.03 ± 0.40

Serum	Protein (G/dL)	6.97 ± 0.09	7.98 ± 0.13	9.99 ± 1.43	9.52 ± 0.39	8.09 ± 0.13
	Cholesterol (mg/dL)	52.04 ± 0.87	64.23 ± 0.45	59.67 ± 0.56	64.99 ± 0.45	61.88 ± 0.54
	VLDL (mg/dL)	23.54 ± 1.09	22.23 ± 1.07	19.55 ± 0.88	26.98 ± 0.75	14.98 ± 0.76
	Tg (mg/dL)	114.24 ± 3.97	110.55 ± 1.34	95.45 ± 0.91	137.35 ± 0.78	76.55 ± 0.77
	Albumin (G/dL)	3.29 ± 0.03	3.45 ± 0.02	3.81 ± 0.02	3.66 ± 0.01	3.10 ± 0.02
	A/G ratio	0.99 ± 0.04	0.68 ± 0.03	0.51 ± 0.02	0.59 ± 0.02	0.64 ± 0.02
	Globulim	3.47 ± 0.23	4.89 ± 0.02	6.50 ± 1.11	5.71 ± 0.19	4.88 ± 0.12

b = *p* < 0.01

c = *p* < 0.05.

**Table 9:** Effect of the test substances on serum testosterone content of control, [ Fe(TAML^1^)(CH_3_COO)_2_] and [ Fe(TAML^2^)(CH_3_COO)_2_] treated, and withdrawal group of rats. Values are mean ± SE.

Group	Serum testosterone (mg/mL)

Control	1.3973 ± 0.076
Fe(TAML^1^)(CH_3_COO)_2_	0.5975 ± 0.002^a^
(50 mg/day/rat for 60 days)	
Withdrawal (30 days)	0.83 ± 0.03
Fe(TAML^2^)(CH_3_COO)_2_	0.675 ± 0.002^a^
(50 mg/day/rat for 60 days)	
Withdrawal (30 days)	1.81 ± 0.039

a = *p* < 0.001.

**Table 10:** Histocytometric data of control, [Fe(TAML^1^)(CH_3_COO)_2_] treated and withdrawal group of rats. Values are mean ± SE.

Tissues	Parameters (*μ*m)	Control	Treated	Withdrawal

	Seminiferous tubule diameter (STD)	240.25 ± 5.02	224.04 ± 5.98	255.00 ± 4.23
Testis	Germinal epithelial cell height (GECH)	92.10 ± 2.70	54.23 ± 2.34^a^	56.02 ± 4.98
	Ledyig cell diameter (LCD)	53.45 ± 2.98	30.00 ± 2.09^a^	42.03 ± 3.98
Cauda epididymis	Epithelial cell height (ECH)	47.50 ± 3.29	21.92 ± 2.57^a^	38.77 ± 4.98
Seminal vesicle	(ECH)	55.02 ± 3.25	50.55 ± 6.24	85.00 ± 10.15
Ventral prostate	(ECH)	37.55 ± 4.78	21.54 ± 3.28^c^	31.05 ± 4.13
Vas deferens	(MLT)	355.24 ± 12.27	328 ± 7.54	475.05 ± 13.28
	(ECH)	50.02 ± 2.40	40.77 ± 3.77^c^	94.05 ± 4.93

a = *p* < 0.001

b = *p* < 0.01.

**Table 11:** Histocytometric data of control, [Fe(TAML^2^)Cl_2_] treatment and withdrawal group of rats. Values are mean ± SE

Tissues	Parameter (*μ*m)	Control	Treated	Withdrawal

	STD	240.45 ± 4.93	105.2 ± 5.9^a^	140.05 ± 5.83
Testis	GECH	88.55 ± 2.79	38.27 ± 2.4^a^	63.10 ± 5.83
	LCH	48.23 ± 3.24	30.55 ± 3.0^b^	45.70 ± 6.73
Cauda epididymis	ECH	47.25 ± 3.77	15.43 ± 2.00^a^	33.90 ± 3.19
	MLT	66.23 ± 4.93	58.55 ± 3.70	63.70 ± 5.92
Seminal vesicle	ECH	56.05 ± 4.67	10.32 ± 1.44^a^	47.45 ± 2.64
Ventral prostate	ECH	39.67 ± 4.60	12.55 ± 1.37^a^	45.40 ± +2.79
Vas deferens	MLT	355.05 ± 30.2	309.55 ± 10.3	374.95 ± 1.67
	ECH	51.98 ± 2.79	32.58 ± 4.7	41.00 ± 2.73

a = *p* < 0.001

b = *p* < 0.01.
